# Concealed urogenital schistosomiasis causing chronic pelvic pain: A case report

**DOI:** 10.1002/ccr3.3654

**Published:** 2020-12-14

**Authors:** Rumbidzai Majangara Karaga

**Affiliations:** ^1^ Department of Obstetrics and Gynaecology University of Zimbabwe College of Health Sciences Harare Zimbabwe

**Keywords:** chronic pelvic pain, diagnostic delay, urogenital schistosomiasis

## Abstract

Urogenital schistosomiasis may mimic or co‐exist with other disease. Clinicians should maintain a high index of suspicion for schistosomiasis in women from endemic areas and travelers to avoid significant morbidity and unnecessary interventions.

## Graphical abstract

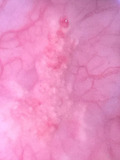

## 1 INTRODUCTION

Schistosomiasis is recognized by the World Health Organization (WHO) as a “neglected tropical disease” of public health concern.^1^ It is endemic in 74 countries especially in Africa and the Middle East. By the year 2001, approximately 85% of the global infections were estimated to be in people from the African continent.^2^ A nationwide cross‐sectional survey in Zimbabwe, conducted from 2010 to 2011, found that the overall prevalence of any schistosomiasis was 22.7%, while heavy infection intensity with any schistosome species was 5.8%.^3^ Schistosoma hematobium was more common than schistosoma mansoni, with an overall prevalence of 18% compared to 7.2%, respectively.^3^

Schistosomiasis is a parasitic infection acquired through skin contact with schistosoma cercariae in fresh water. These migrate through the circulation to the portal system in the liver where they mature into adult worms. Adult worms migrate to mesenteric venules of the bowel and vesical venous plexus where they settle and lay eggs. These eggs migrate into small venules and subsequently epithelial surfaces within the urogenital system. Female genital schistosomiasis (FGS) is characterized by the presence of worms and eggs in the genital tract. The host immune response to the presence of schistosome eggs is responsible for the clinical manifestation. The infestation typically lasts 5‐10 years but may progress to 30 years.^4^ FGS is most frequently caused by Schistosoma hematobium, but there are rare reports of FGS being caused by S. mansoni, Schistosoma japonicum, and Schistosoma intercalatum.^5^ The uterine cervix is the commonest site of FGS followed by the uterine body, adnexa, vagina, and vulva.^6^

The clinical presentation of FGS is nonspecific and may mimic other genitourinary disease such as sexually transmitted infections, pelvic inflammatory disease, and cystitis from other infective or inflammatory causes.^7^ Some women develop ulcerative or fibrotic granulomas on the genital organs that resemble malignancy. FGS has been associated with infertility, ectopic pregnancies, miscarriages, and anemia.^7^

This report discusses urogenital manifestations of schistosomiasis in a woman without obvious exposure to infested fresh water, and without typical findings of hematuria and schistosome eggs on microscopic examination of the urine. It highlights the importance of maintaining a high index of suspicion for schistosomiasis in endemic areas and in individuals at risk.

## 2 CASE REPORT

The report describes a 32‐year‐old woman who presented to an emergency department with severe acute‐on‐chronic pelvic pain for 4 days and inability to walk straight in the preceding 24 hours. There was associated dysuria, frequency of micturition, copious whitish nonfoul smelling per vaginal discharge, and anorexia. She was passing stool normally.

She reported a history of chronic pelvic pain and intermittent dysuria for over 10 months which she noticed early in pregnancy. At approximately 20 weeks of gestation, preterm prelabor rupture of membranes (PPROM) occurred. She was admitted in hospital most of the time until antepartum hemorrhage occurred culminating in preterm delivery of a live baby at 32 weeks of gestation. She reported having received several courses of antibiotic therapy during the hospitalization in pregnancy. Source records to determine the investigations undertaken or their results nor the names of antibiotics given during pregnancy were not available because care had been in a different hospital in a different part of the country and she did not possess any documentation. Symptoms had persisted postpartum until the current presentation.

She denied suffering from or being treated for a sexually transmitted infection earlier. She had one sexual partner and did not have evidence or suspicion that her husband had other sexual partners. The patient was not on treatment for any other chronic medical conditions and had not had any other surgeries besides the cesarean section.

She had a degree in social work but was unemployed. She did not smoke, drink alcohol, or use other recreational drugs. She was the only wife to a 36‐year‐old husband, but they were now estranged following the prolonged hospital admissions, piling medical bills, and ongoing pelvic pain which made sexual intercourse uncomfortable. She had always lived in urban areas, mostly in the capital city, and used modern methods of sanitation. She denied using or swimming in unprotected water sources.

On examination, she was fully conscious with no clinical signs of anemia. Her vital signs were normal, blood pressure of 113/69 mm Hg, pulse rate of 69 beats/min, and temperature of 36.3°C. The neurological, cardiovascular, and respiratory systems were normal. The abdomen was flat, with significant guarding and rebound tenderness in the lower abdomen. On pelvic examination, there was a moderate creamish cervico‐vaginal discharge. The cervical os was closed, and the uterus was not enlarged. There was cervical motion tenderness and bilateral adnexal tenderness.

Urine and a high vaginal swab were sent for microscopy, culture, and sensitivity (mcs). The full blood count and renal function tests were normal. The serum quantitative human chorionic gonadotropin was 0.14 IU/L. Ultrasound scan examination of the pelvis and abdomen showed a normal uterus and adnexa, with no free pelvic fluid. The urinary bladder had a normal mucosal outline. The kidneys, ureter, appendix, bowel, liver, gall bladder, spleen, and pancreas all had normal outlines.

The preliminary diagnosis was severe pelvic inflammatory disease. She was treated with a maintenance infusion of normal saline 1 L to run every eight hours and intravenous antibiotic therapy viz, ceftriaxone 1 g twice daily, gentamycin 240 mg once daily, and metronidazole 500 mg thrice daily.

**FIGURE 1:**
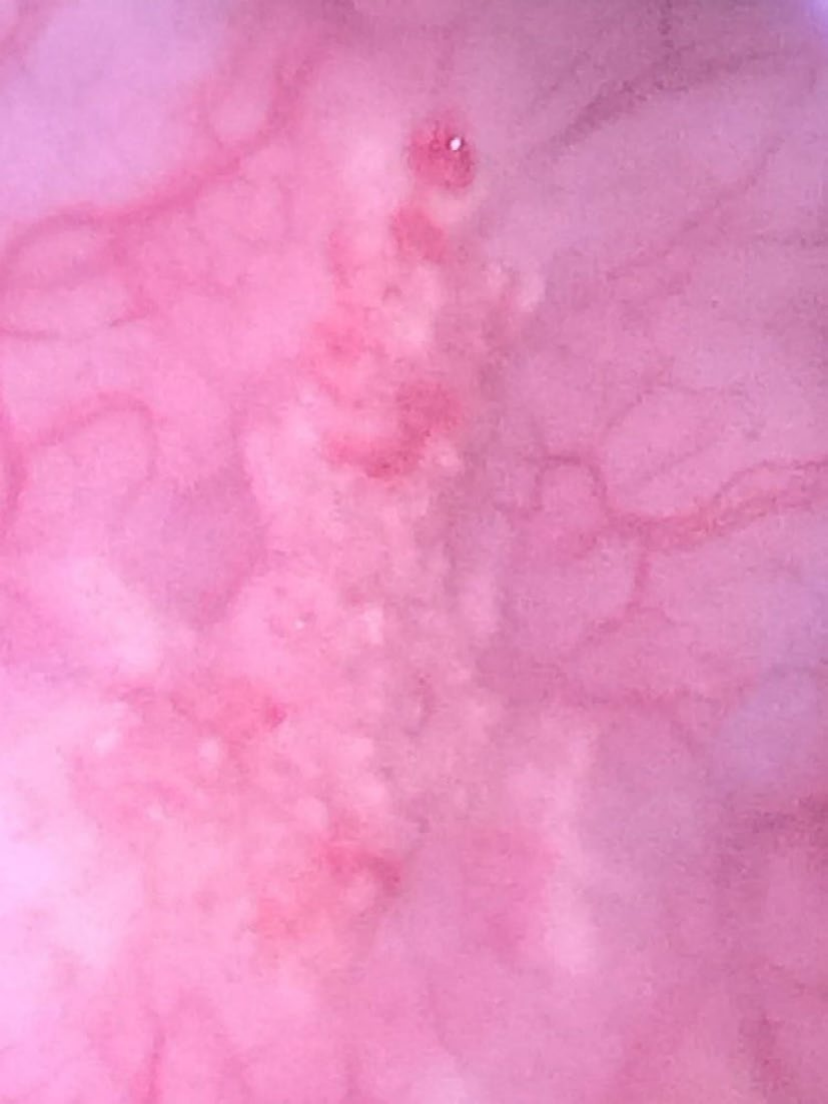
Cystoscopy showing sandy mass

The patient remained apyrexial for 48 hours but her symptoms and physical examination findings did not improve. A general surgical opinion was sought and a diagnostic laparoscopy and cystoscopy were performed. At laparoscopy, a 2 cm portion of omentum was adherent to the anterior abdominal wall, below the umbilicus. The omentum was released easily with traction and use of bipolar coagulation. The uterus, fallopian tubes, ovaries, appendix, bowel, and liver surface appeared normal. There were no other significant findings to account for her symptoms. At cystoscopy, a sandy mass was located on the right lateral wall of the bladder mucosa (Figure 1).

A biopsy of the mass which was loosely adherent to the mucosa was taken and sent for histological analysis.

Urine and high vaginal swab mcs results were available immediately postoperatively. Vaginal microscopy showed positive cocci and positive rods resembling lactobacilli, while a moderate growth of staphylococcus was observed after 48 hours of incubation. There were no significant findings on urine microscopy, but culture showed 10^4^ ‐ 10^5^ colony forming units/ml of *Staphylococcus aureus*. *Staphylococcus aureus* cultured from both urine and vaginal swab were resistant to multiple antibiotics, viz; ampicillin, augmentin, cefuroxime, ciprofloxacin, clindamycin, cloxacillin, cotrimoxazole, erythromycin, tetracycline, meropenem, and teicoplanin. It was sensitive only to vancomycin. Treatment with vancomycin 500 mg (slow infusion to run over 60 minutes) every 6 hours was commenced. However, she developed severe generalized pruritis halfway through administration of the first dose. She was hemodynamically stable and did not have difficulty breathing or a skin rash. The infusion and any further doses were discontinued. Single‐dose hydrocortisone 200 mg intravenously and promethazine 25 mg intramuscularly were administered. She remained hemodynamically stable and the pruritis resolved by end of the day. Microbiological specialists were consulted and they advised to withhold vancomycin until after a repeat urine mcs evaluation and testing for oxacillin sensitivity. Ceftriaxone, gentamycin, and metronidazole were continued at the preoperative doses. She began mobilizing unaided within 48 hours postoperatively and reported that her symptoms were improving.

Repeat urine mcs did not grow *S aureus*. There was a profuse growth of Klebsiellae pneumoniae sensitive to ciprofloxacin, amikacin, and carbapenems. It was resistant to cefuroxime, ceftriaxone, gentamycin, ampicillin, amoxicillin, amoxyclavulanic acid, piperacillin, tazobactam, and gentamicin. There was also a profuse growth of candida albicans.

Intravenous antibiotics were withheld. Treatment with ciprofloxacin 500 mg twice daily for five days and miconazole vaginal cream was commenced. She was discharged on day eight after admission. Though improved, she still complained of pelvic pain.

She presented for review after 2 weeks with vomiting, and worsening dysuria and pelvic pain. She was readmitted and managed as a case of chronic cystitis/pyelonephritis. Empiric therapy with meropenem 500 mg thrice daily was commenced.

Histology results were followed up and showed the presence of schistosoma, but the species was not reported. Praziquantel 2400 mg single dose was given, and meropenem was withheld 72 hours after commencement. She was subsequently discharged and recovered uneventfully.

She was reviewed telephonically at 3 months and 2 years and reported to be well with no urogenital symptoms.

## 3 DISCUSSION

The report described a 32‐year‐old woman who had a prolonged history of pelvic pain and urogenital symptoms associated with significant bacteriuria. The symptoms persisted despite multiple courses of antibiotic therapy. A diagnosis of schistosomiasis was made after diagnostic laparoscopy, cystoscopy, and bladder biopsy. Complete recovery occurred after treatment with praziquantel.

There was a significant delay in diagnosis due to the nonidentification of Schistosoma ova during microscopic analysis of the urine of the patient despite this being done on at least two samples. This is a common phenomenon.^8^, ^9^ Up to 53% of women with visible FGS lesions had no schistosome ova observed in urine or stool microscopy in rural Zimbabwe.^9^

Previously, the gold standard for diagnosis of FGS was microscopic examination of the crushed biopsy of genital tissue.^10^ Having genital schistosomiasis is a stand‐alone risk factor for human immunodeficiency virus (HIV) infection due to recruitment of cluster of Differentiation 4 positive (CD4+) immune cells into the inflamed genital tissue. Genital tract biopsy is invasive and further predisposes to HIV acquisition should the woman have intercourse before the site completely heals.^6^ The WHO currently recommends that the diagnosis of FGS be based on visual inspection which can be improved by a digital camera or colposcope.^7^ Characteristic features of FGS on visual inspection are “sandy patches appearing as single or clustered grains or homogenous yellow areas, or rubbery papules”.^7^ Lesions typically exhibit negative reactions to acetic acid and Lugol's iodine. They are not confined to the transformation zone and may be accompanied by abnormal vascularization and inflammation.^11^ No suspicious lesions were visualized with the naked eye in this patient. Since the characteristic lesions were visualized at cystoscopy, colposcopy was not necessary.

The sensitivity for diagnosing FGS is low using the following laboratory methods, pap smear (15%), and polymerase chain reaction for schistosoma in vaginal lavage fluid (53%‐67%).^6^ Serologic tests diagnose exposure but do not differentiate active from inactive infection and may be falsely negative in the acute phase of infection.^12^

Schistosomiasis has been associated with chronic urinary infections and septicemia caused by enterobacteriacae.^13^ This phenomenon is thought to be due to schistosome worms being foci of bacterial multiplication and evasion of host immune responses. Schistosoma may also depress the immune response, such that eradication of bacterial sepsis may be achieved only after concomitant use of antibiotics and praziquantel.^13^ Similar mechanisms may explain the prolonged symptomatology in this patient.

A single dose of praziquantel (40 mg/kg) has high potency against all forms of schistosoma infection.^7^ It is cheap and widely available. However, praziquantel is not protective against new infections, nor does it reverse existing lesions caused by schistosomiasis. Since benefits outweigh any potential risks in endemic areas, the WHO recommends regular praziquantel preventive chemotherapy for children and women, including during pregnancy and lactation.^14^ No diagnosis is required in areas of high endemicity, while screening is preferred in areas of low endemicity.^14^

Multi‐drug‐resistant *S. aureus* was not cultured, from the urine, on repeat testing after the single dose of vancomycin. Single‐dose vancomycin is inadequate therapy for the eradication of multi‐drug‐resistant *S. aureus* since it is typically given as multiple doses.^15^ It is possible that the first sample had been contaminated. The patient most likely experienced “red man syndrome” (RMS) instead of anaphylaxis because the pruritis was isolated. Severe pruritis is known to occur as part of the RMS, an idiopathic infusion reaction to parenteral vancomycin which is not immunoglobulin‐E mediated. It is usually associated with an infusion rate faster than the recommended rate of no more than 10 mg/min.^16^ The use of infusion pumps facilitates controlled infusion rates. In resource limited settings, infusion pumps are not readily available such that this patient may have had an unintentional fast rate of infusion which precipitated pruritis.

Although FGS has been associated with obstetric complications such as ectopic pregnancies and miscarriages, some researchers have not found any association with preterm birth.^17^, ^18^ Concomitant bacterial urinary tract infection may have predisposed her to PPROM and preterm delivery.^19^ Although there were no source records available to prove bacteriuria during pregnancy, she had symptoms suggestive of infection of the urogenital system.

Schistosomiasis can be eradicated through mass drug administration programs and vector control measures such as improved water and sanitation and snail control.^1^

## 4 CONCLUSION

Urogenital schistosomiasis continues to evade diagnosis even in endemic areas. When managing women who live in or have travelled to endemic areas, it is important to maintain a high index of suspicion for schistosomiasis even in the presence of concomitant disease or urine microscopy negative for schistosoma. This averts significant morbidity and unnecessary interventions including radical surgery.

## CONFLICT OF INTEREST

None declared.

## AUTHOR CONTRIBUTIONS

RM: case management, data collection, and preparing the manuscript.

## ETHICAL APPROVAL

Written informed consent was obtained from the patient for publication of this case report and the accompanying images.

## Data Availability

All relevant clinical data and images are included in this report. Any additional information is available from the author following reasonable request.

## References

[1] World Health Organization . Schistosomiasis: Progress Report 2001–2011, Strategic Plan 2012–2020. World Health Organization; 2013. https://apps.who.int/iris/handle/10665/78074. Accessed July 9, 2020.

[2] Chitsulo L , Engels D , Montresor A , Savioli L . The global status of schistosomiasis and its control. Acta Trop. 2000;77(1):41‐51. 10996119 10.1016/s0001-706x(00)00122-4 PMC5633072

[3] Midzi N , Mduluza T , Chimbari MJ , et al. Distribution of schistosomiasis and soil transmitted helminthiasis in Zimbabwe: towards a national plan of action for control and elimination. PLoS Negl Trop Dis. 2014;8(8):e3014. 25121489 10.1371/journal.pntd.0003014 PMC4133179

[4] Hornstein L , Lederer G , Schechter J , et al. Persistent *Schistosoma mansoni* infection in Yemeni immigrants to Israel. Isr J Med Sci. 1990;26(7):386‐389. 2117600

[5] Christinet V , Lazdins‐Helds JK , Stothard JR , Reinhard‐Rupp J . Female genital schistosomiasis (FGS): from case reports to a call for concerted action against this neglected gynaecological disease. Int J Parasitol. 2016;46(7):395‐404. 27063073 10.1016/j.ijpara.2016.02.006

[6] Kjetland EF , Leutscher PDC , Ndhlovu PD . A review of female genital schistosomiasis. Trends Parasitol. 2012;28(2):58‐65. 22245065 10.1016/j.pt.2011.10.008

[7] World Health Organisation . Female Genital Schistosimiasis. WHO; 2015. https://apps.who.int/iris/bitstream/handle/10665/180863/9789241509299_eng.pdf;jsessionid=664739BEF05A88DFF1278C0BFCAADC2B?sequence=1. Accessed June 16, 2020.

[8] Poggensee G , Kiwelu I , Saria M , Richter J , Krantz I , Feldmeier H . Schistosomiasis of the lower reproductive tract without egg excretion in urine. Am J Trop Med Hyg. 1998; 59(5):782‐783. 9840597 10.4269/ajtmh.1998.59.782

[9] Kjetland EF , Ndhlovu PD , Mduluza T , et al. Simple clinical manifestations of genital *Schistosoma haematobium* infection in rural Zimbabwean women. Am J Trop Med Hyg. 2005;72(3):311‐319. 15772328

[10] Poggensee G , Kiwelu I , Weger V , et al. Female genital schistosomiasis of the lower genital tract: prevalence and disease‐associated morbidity in Northern Tanzania. J Infect Dis. 2000;181(3):1210‐1213. 10720558 10.1086/315345

[11] Norseth HM , Ndhlovu PD , Kleppa E , et al. The colposcopic atlas of schistosomiasis in the lower female genital tract based on studies in Malawi, Zimbabwe, Madagascar and South Africa. PLoS Negl Trop Dis. 2014;8(11):e3229. 25412334 10.1371/journal.pntd.0003229 PMC4238986

[12] Rabello ALT , Garcia MMA , Pinto da Silva RA , Rocha RS , Katz N . Humoral immune responses in patients with acute *Schistosoma mansoni* infection who were followed up for two years after treatment. Clin Infect Dis. 1997;24(3):304‐308. 9114177 10.1093/clinids/24.3.304

[13] Chieffi PP . Interrelationship between schistosomiasis and concomitant diseases. Mem Inst Oswaldo Cruz. 1992;87(Suppl 4):291‐296. 1343911 10.1590/s0074-02761992000800045

[14] Crompton DWT , World Health Organization . Preventive Chemotherapy in Human Helminthiasis: Coordinated Use of Anthelminthic Drugs in Control Interventions: A Manual for Health Professionals and Programme Managers. World Health Organization; 2006 https://apps.who.int/iris/handle/10665/43545. Accessed July 14, 2020.

[15] Liu C , Bayer A , Cosgrove SE , et al. Clinical practice guidelines by the infectious Diseases Society of America for the treatment of methicillin‐resistant *Staphylococcus aureus* infections in adults and children. Clin Infect Dis. 2011;52(3):e18‐e55. 21208910 10.1093/cid/ciq146

[16] Sivagnanam S , Deleu D . Red man syndrome. Crit Care. 2003;7(2):119. 12720556 10.1186/cc1871 PMC270616

[17] Murenjekwa W , Makasi R , Ntozini R , et al. Determinants of urogenital schistosomiasis among pregnant women and its association with pregnancy outcomes, neonatal deaths, and child growth. J Infect Dis;jiz664. https://academic.oup.com/jid/advance‐article/doi/10.1093/infdis/jiz664/5674952. Accessed July 14, 2020 10.1093/infdis/jiz664 PMC8064048 31832636

[18] Mombo‐Ngoma G , Honkpehedji J , Basra A , et al. Urogenital schistosomiasis during pregnancy is associated with low birth weight delivery: analysis of a prospective cohort of pregnant women and their offspring in Gabon. Int J Parasitol. 2017;47(1):69‐74. 28003151 10.1016/j.ijpara.2016.11.001

[19] Smaill FM , Vazquez JC . Antibiotics for asymptomatic bacteriuria in pregnancy. Cochrane Database Syst Rev. 2019;2019(11):CD000490. 10.1002/14651858.CD000490.pub4 PMC6953361 31765489

